# Long-term outcomes of nasopharyngeal carcinoma treated with helical tomotherapy using simultaneous integrated boost technique: A 10-year result

**DOI:** 10.3389/fonc.2022.1083440

**Published:** 2023-01-20

**Authors:** Lingling Meng, Feng Teng, Qiteng Liu, Lei Du, Boning Cai, Chuanbin Xie, Hanshun Gong, Xinxin Zhang, Lin Ma

**Affiliations:** ^1^ Medical School of the Chinese People’s Liberation Army (PLA), Beijing, China; ^2^ Department of Radiation Oncology, First Medical Center of Chinese PLA General Hospital, Beijing, China; ^3^ Department of Radiation Oncology, China-Japan Friendship Hospital, Beijing, China; ^4^ Department of Radiation Oncology, Beijing Luhe Hospital, Affiliated to Capital Medical University, Beijing, China; ^5^ Department of Radiation Oncology, Hainan Hospital of the Chinese PLA General Hospital, Sanya, China; ^6^ Department of Otorhinolaryngology, First Medical Center of Chinese PLA General Hospital, Beijing, China

**Keywords:** nasopharyngeal carcinoma, helical tomotherapy (HT), survival, chemotherapy, radiotherapy

## Abstract

**Background:**

To evaluate the long-term survival and treatment-related toxicities of helical tomotherapy (HT) in nasopharyngeal carcinoma (NPC) patients.

**Methods:**

One hundred and ninety newly diagnosed non-metastatic NPC patients treated with HT from September 2007 to August 2012 were analyzed retrospectively. The dose at D95 prescribed was 70-74Gy, 60-62.7Gy and 52-56Gy delivered in 33 fractions to the primary gross tumor volume (pGTVnx) and positive lymph nodes (pGTVnd), the high risk planning target volume (PTV1), and the low risk planning target volume (PTV2), respectively, using simultaneous integrated boost technique. The statistical analyses were performed and late toxicities were evaluated and scored according to the Common Terminology Criteria for Adverse Events (version 3.0).

**Results:**

The median follow-up time was 145 months. The 10-year local relapse-free survival (LRFS), nodal relapse-free survival (NRFS), distant metastasis-free survival (DMFS) and overall survival (OS) were 94%, 95%, 86%, and 77.8%; respectively. Fifty (26.3%) patients had treatment-related failures at the last follow-up visit. Distant metastasis, occurred in 25 patients, was the major failure pattern. Multivariate analysis showed that age and T stage were independent predictors of DMFS and OS, Concomitant chemotherapy improved overall survival, but anti-EGFR monoclonal antibody therapy failed. The most common late toxicities were mainly graded as 1 or 2.

**Conclusions:**

Helical tomotherapy with simultaneous integrated boost technique offered excellent long-term outcomes for NPC patients, with mild late treatment-related toxicities. Age and clinical stage were independent predictors of DMFS and OS. And, concurrent chemotherapy means better OS. Further prospective study is needed to confirm the superiority of this technology and to evaluate the roles of anti-EGFR monoclonal antibody treatment.

## Introduction

Nasopharyngeal carcinoma (NPC) is the most common malignant tumor of the head and neck in China, about 70% ~ 80% of patients are combined with the neck lymph node metastasis, and 10% ~ 15% combined with distant metastases at the first diagnosis (1). Radiation therapy is the only curative treatment method for non-metastatic NPC, and intensity-modulated radiation therapy (IMRT) has been accepted as the standard radiation technique (2). The addition of chemotherapy and anti-EGFR monoclonal antibody (Mab) treatment improves the local control rate (LCR) and the overall survival rate (OS) for advanced NPC patients (3, 4).

Helical tomotherapy (HT) is an emerging IMRT technique that mounts a 6-MV linear accelerator on a ring frame around the accelerator bed (5). The couch passes axially through the center of the stand as it rotates to irradiate the target area. With the capacity to deliver highly conformal dose distribution and pretreatment setup verification (6), HT has achieved better results in the treatment of head and neck cancer (7). In September 2007, our center installed the first HT device in China and the initial observation showed that 3-year local relapse-free survival, nodal relapse-free survival and distant metastasis-free survival were more than 90%, and 3-year overall survival was more than 85% for NPC patients (8). Here, we present a retrospective analysis of long-term (10-year) outcomes and late toxicities of HT in 190 patients with NPC.

## Materials and methods

### Patient’s characteristics

One hundred and ninety NPC patients, treated with Hi Art TomoTherapy system (Accuray, America) at our center between September 2007 and August 2012, were analyzed. All patients underwent nasopharyngeal and skull base computed tomography (CT) or magnetic resonance imaging (MRI), endoscopic evaluation, complete blood count, liver and kidney function tests, neck and abdomen ultrasound, and bone scan. Positron emission tomography (PET) is optional. The clinical stage was determined according to the UICC 2002 staging system. **Table 1** summarizes patient characteristics. This study was conducted in accordance with the declaration of Helsinki. This study was conducted with approval from the Ethics Committee of the Chinese PLA General Hospital. Written informed consent was obtained from all participants.

**Table 1:** Patient’s characteristics.

Characteristics	Patients	
	Number	%
Age (median)	10-81(44)	
Male	144	75.8
Female	46	24.2
ECOG performance status		
0	57	30
1	113	59.5
2	20	10.5
UICC 2002 stage		
I	16	8.4
II	64	33.7
III	71	37.4
IV	39	20.5

### Treatment

The definition of the targets, techniques of planning and the HT delivery were described previously (2). Briefly, the planning dose at D95 was prescribed to the gross tumor volume (pGTVnx) and positive lymph nodes (pGTVnd) at 70–74Gy, the high risk planning target volume (PTV1) at 60–62.7 Gy and the low risk planning target volume (PTV2) at 52–56 Gy, respectively, in 33 fractions. The irradiation was delivered once daily, 5 days per week. MVCT imaging was performed prior to each part of HT treatment to validate patient settings during HT treatment. MVCT image-guidance is the automatic and manual registration of MVCT images and planned CT images, based on bone and tissue anatomy.

Cisplatin-based chemotherapy with or without concomitant anti-EGFR Mab treatment was given to 129 patients, concomitant anti-EGFR Mab treatment without chemotherapy was given to 30 patients, and radiotherapy alone was given to 31 patients. Neoadjuvant chemotherapy includes 1–2 cycles of DP (docetaxel 75 mg/m^2^, d1, cisplatin 80 mg/m^2^, d1 and every 3 weeks) or a single DDP regimen. According to clinical stages, tolerance and economic status, concurrent chemotherapy and/or anti-EGFR Mab therapy were performed in one of the four modes: 1) cisplatin 80 mg/m^2^, d1, every 3 weeks; 2) docetaxel 60 mg/m^2^, d1 and cisplatin 60 mg/m^2^, every 3 weeks; 3) cetuximab 250 mg/m^2^ or nimotuzumab 200 mg, d1, every week; 4) cetuximab 250 mg/m^2^ or nimotuzumab 200 mg, d1, every week and cisplatin 80mg/m^2^, d1, every 3 weeks. Adjuvant chemotherapy consisted of 4~6 cycles of DP program. Each patient received no more than 6 cycles of chemotherapy (including neoadjuvant, concurrent and adjuvant modes).

### Follow-up and evaluation of outcomes

Acute side-effects were investigated weekly and peak toxicities were recorded and graded according to the established RTOG/EORTC criteria and the Common Terminology Criteria for Adverse Events (Version 3.0) as described previously (8). Patients’ follow-up examinations were conducted 1 month after the completion of radiotherapy, and then every 3 months for the first year, every 6 months in the second year, and annually thereafter. Late toxicity were evaluated and scored according to the Common Terminology Criteria for Adverse Events version 3.0 (9) (CTCAE 3.0).

### Statistical analysis

The Kaplan-Meier method was used to analyze local recurrence-free survival (LRFS), lymph node recurrence-free survival (NRFS), distant metastasis-free survival (DMFS) and overall survival (OS). Different prognostic factors were analyzed by log-rank test and multivariate analysis was performed using Cox proportional hazards model. P<0.05 was considered significant. Statistical analyzes were performed using the SPSS software package (Version 22.0, SPSS Inc., an IBM Company; Chicago, IL, USA).

## Results

### Treatment outcomes

Case selection, follow-up and statistical analysis were done by two different researchers to ensure the accuracy of the data. The median follow-up was 145 months, ranging from 141 to 148 months from the start of radiation therapy. The median age of the patients was 44 years (range 10-81 years), and the ratio of females (n=46) to males (n=144) was 1:3. The details were shown in **Table 1**.

**Figure 1:**
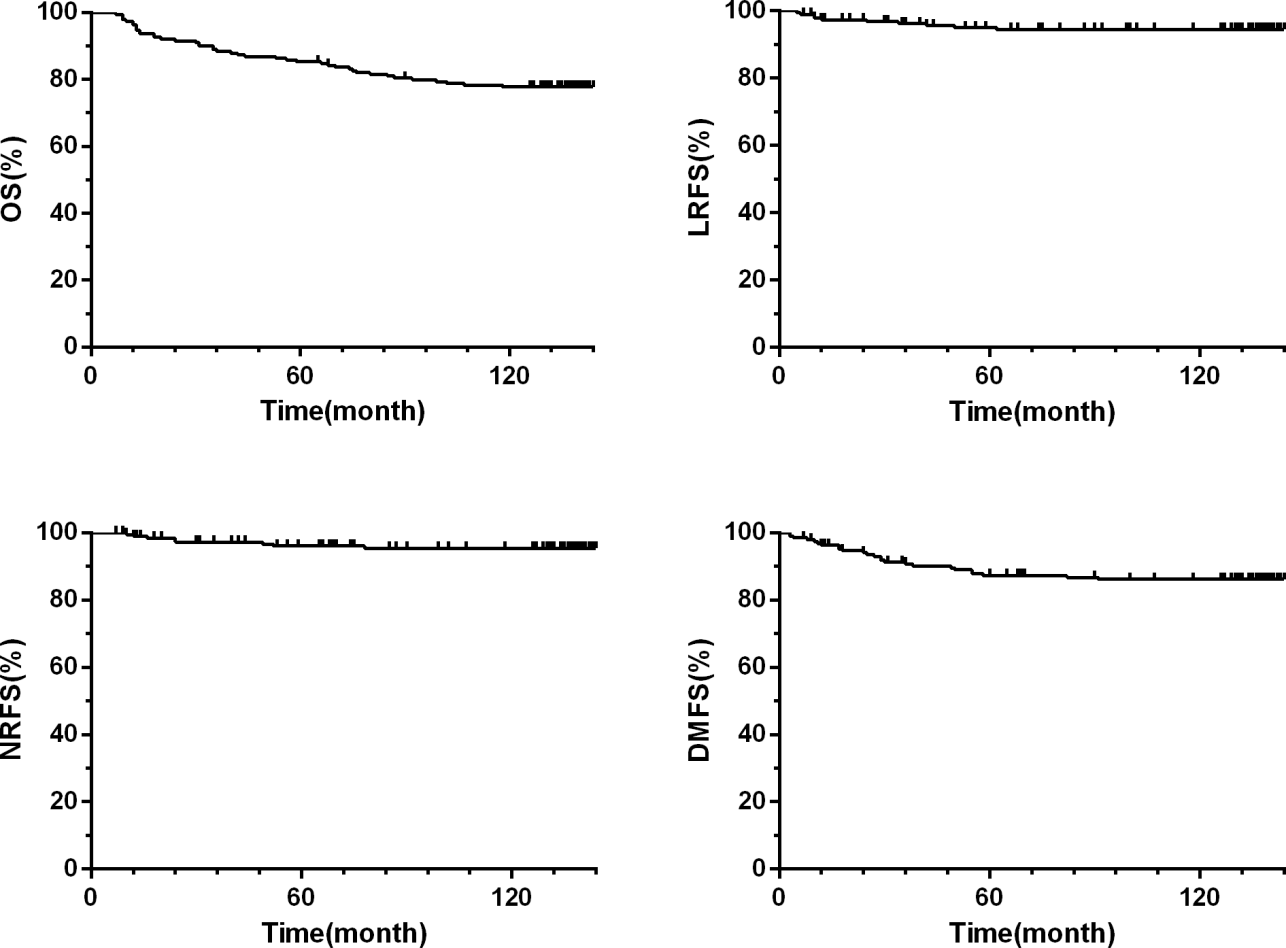
Kaplan-Meier estimate of local relapse-free survival (LRFS), nodal relapse-free survival (NRFS), distant metastases-free survival (DMFS), and overall survival (OS) rates.

The 10-year LRFS, NRFS, DMFS, and OS were 94.0%, 95%, 86% and 77.8%, respectively. The 10-year OS and NRFS for patients with stage T1/2 and stage T3/4 were 81% vs.67% and 99%vs. 88%, respectively (c= 3.9, *p*= 0.01 and c= 10, *p*= 0.01). The 10-year LRFS, NRFS, DMFS and OS for patients with stage I/II were better than those with stage III/IV(*p<* 0.05). The 10-year NRFS and DMFS for patients with N0-1 were better than those with N2-3 (*p<* 0.05). No significant differences were observed in OS and LRFS, between the patients with N0-1 and N2-3 (*p*> 0.05) (**Table 2**; **Figure 1**).

**Table 2:** Characteristics of 190 patients and univariate analysis of prognostic factors.

Characteristics	No. of patients	10-y LRFS (%)	P value	10-y NRFS (%)	P value	10-y DMFS (%)	P value	10-y OS (%)	P value
Sex									
Female	46	95	0.67	95	0.97	83	0.047	75	0.128
Male	144	93		95		95		87	
Age (y)									
<30	22	91	0.56	95	0.71	80	0.056	77.3	0.001
30-65	154	95		96		83		81.2	
≥65	14	96		86		59		42.9	
T classification									
T1-2	121	96.5	0.093	99	0.001	0.89	0.146	84.2	0.004
T3-4	69	90.5		88		0.82		66.7	
N classification									
N0	39	94.9	0.867	97	0.003	92	0.021	82.1	0.394
N1	69	94.2		98		89		79.7	
N2	70	94.3		95		85		77.1	
N3	12	91.2		73		55		58.3	
Concurrent chemotherapy									
Yes	89	94	0.885	96	0.894	86	0.959	82	0.21
No	101	95		95		84		73	
Anti-EGFR Mab treatement									
Yes	81	96	0.392	96	0.7	81	0.082	86	0.756
No	109	93		95		90		82	
Clinical stage									
I-II	75	98	0.041	99	0.015	95	0.007	89	0.002
III-IV	115	92		92		80		70	

Until the last follow-up, treatment failure was observed in 50 patients. Among these patients, 12 and 8 patients developed local and nodal failure, respectively, and distant metastasis occurred in 25 patients was the major failure pattern after treatment. Lung, bone and liver were the most common metastatic organs. Among the 50 failed patients, 6 patients died of local failure or regional failure and 20 patients died of multiple organ failure. In addition, Five patients (stage T3-4) died of primary pharyngeal vessel bleeding after the completion of radiotherapy, and 6 died of massive hemorrhage 1 year after radiotherapy. One patient died of cerebral hernia 9 months after the completion of radiotherapy. Otherwise, 2 patients died of other causes, one from systemic lupus erythematosus and one from gas poisoning. (**Table 3**).

**Table 3:** Pattern of failures and cause of deaths.

Variable	No. of patients
Pattern of failure	
Distant metastasis	25
Local and/or regional failures	18
Local failure alone	10
Regional failure alone	6
Local and regional failures	2
Distant and local/regional failures	4
Total	50
Cause of death	
Distant metastasis	20
Local or regional failure	6
Radiation-related complications	11
Other malignant tumors	1
Non-cancer causes	2
Unknown causes	2
Total	42

### Prognostic factors

The potential prognostic factors for OS, LRFS, NRFS, and DMFS included gender, age, T stage, N stage, and various chemotherapy and anti-EGFR Mab treatment patterns. Univariate analysis by log-rank test showed that age and N stage were significantly associated with DMFS, age and T stage were significantly associated with OS, T stage and N stage were significantly associated with NRFS, and clinical stage was significantly associated with LRFS, NRFS, DMFS and OS. (**Table 2**).

**Figure 2:**
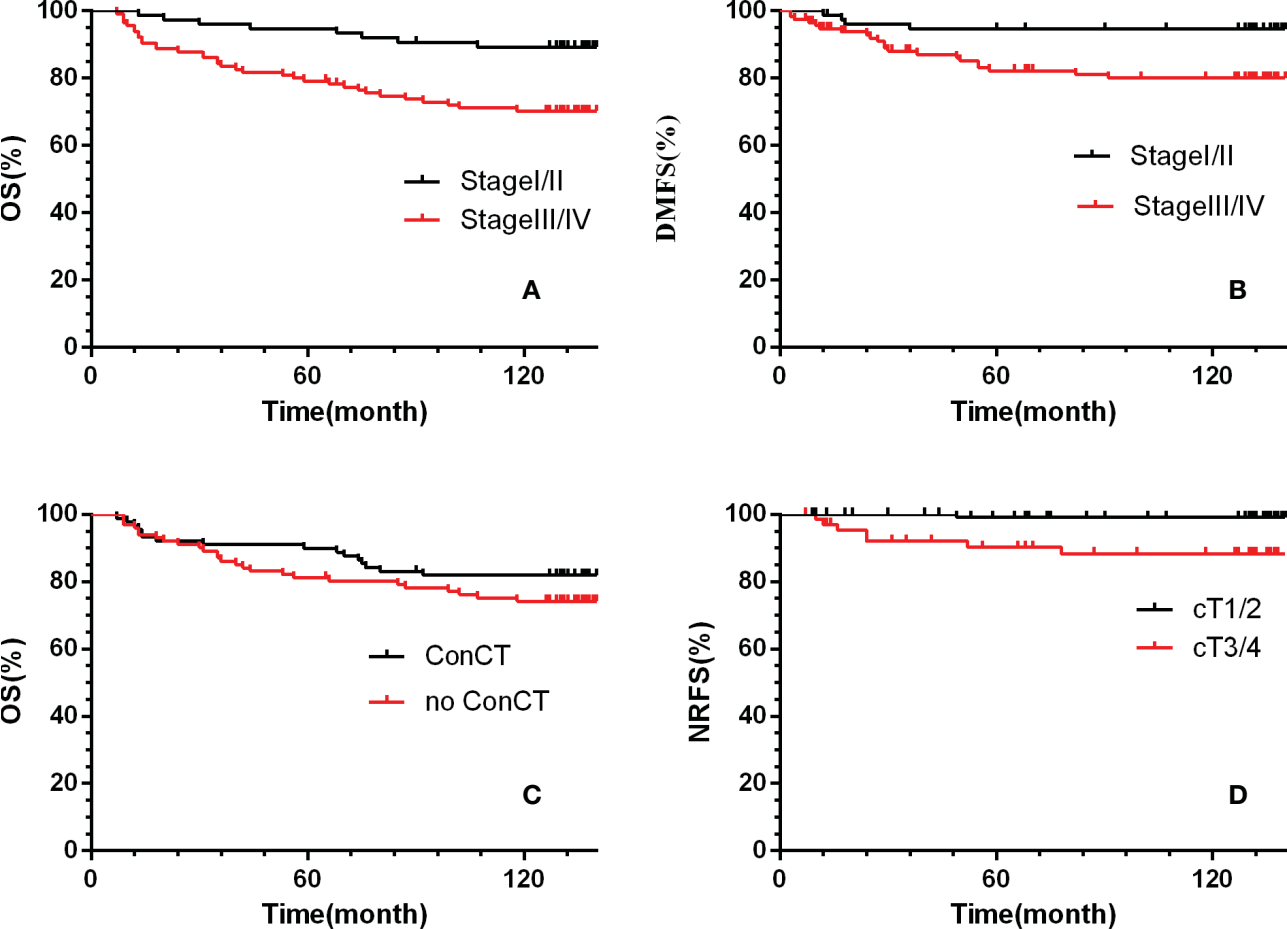
Kaplan-Meier survival curves for the two treatment groups. **(A, C)** overall survival, **(B)** distant metastasis-free survival, **(D)** nodal relapse-free survival. ConCT=concurrent chemotherapy.

Multivariate analysis by Cox proportional hazards model showed that only the T stage was an independent predictor for NRFS (HR=13.9, 95%CI 1.72–5.2, *p*= 0.01).The clinical stage was an independent predictor for LRFS, DMFS and OS. And, age and concurrent chemotherapy were independent predictors for OS. (**Table 4**; **Figure 2**).

**Table 4:** Multivariate analysis of prognostic factors for 190 patients.

	LRFS		NRFS		DMFS		OS	
Characteristics	HR (95%CI)	P value	HR(95%CI)	P value	HR (95%CI)	P value	HR (95%CI)	P value
Sex								
Male vs. female	1.25 (0.31 - 5.15)	0.75	1.28 (0.23 - 7.04)	0.774	0.29 (0.7 - 1.3)	0.09	0.5 (0.2-1.2)	0.121
Age (y)								
<30 vs. 30-65 vs. ≥65	8.1 (0.5 - 19.1)	0.96	0.214 (0.012 - 3.65)	0.28	0.21 (0.05 - 0.90)	0.01	3.1 (1.4-6.9)	0.005
T classification								
T1-2 vs. T3-4	0.7 (0.1 - 4.2)	0.7	13.9 (1.71 - 5.2)	0.01	0.95 (0.38 - 2.4)	0.92	1.26 (0.53-3.01)	0.59
N classification								
N0-1 vs. N2-3	0.13 (0.01 - 1.3)	0.08	3.01 (0.54 - 8.8)	0.21	0.74 (0.22 - 2.5)	0.62	0.82 (0.35-1.89)	0.63
Concurrent chemotherapy								
Yes or No	0.6 (0.14 - 2.44)	0.47	0.326 (0.06 - 1.6)	0.171	0.62 (0.26 - 1.5)	0.28	0.42 (0.21-0.82)	0.01
Anti-EGFR Mab treatement								
Yes or No	0.37 (0.08 - 1.66)	0.19	0.51 (0.10 - 2.49)	0.4	1.33 (0.57 - 3.1)	0.51	0.75 (0.4-1.41))	0.38
Clinical stage								
I+II vs. III+IV	6.4 (1.81 - 9.1)	0.02	4.8 (0.5 - 5.4)	0.96	3.9 (1.3 - 11.4)	0.01	5.5 (1.57-13.9)	0.01

### Late toxicities

The acute treatment-related toxicities were described previously (8). At the last follow-up visit, 139 patients could be evaluated for the late treatment-related toxicities, with 51 patients lost or died. Grade1~2 xerostomia, subcutaneous tissue fibrosis, hearing loss, and tooth sensitivity were the most common late toxicities. Two patients had loss of tooth, and 2 others had pulpitis and treated with surgical operation. No toxicity and side effects of grade 3 or above trismus, temporal lobe necrosis, cranial nerve palsy, and eyeball injury were found. The data of late treatment-related toxicities were listed in **Table 5**.

**Table 5:** Late radiation related toxicities of 139 patients.

Late complications	No.of patients	
	Grade I/II	Grade III
Xerostomia	72	1
Hearing impairment	43	3
Subcutaneous fibrosis	63	10
Trismus	4	0
Temporal lobe necrosis	10	0
Cranial nerve palsies	34	0
Eyeball damage	22	0

## Discussion

Intensity-modulated radiotherapy (IMRT) has been demonstrated to be potentially less toxic and more effective than conventional radiation techniques, even achieved a survival benefit compared with three-dimensional conformal radiotherapy 3DCRT (10). The advent of helical tomotherapy (HT) as image-guided radiotherapy (IGRT) has offered the potential of improved target conformation and sparing of critical structures (11). HT is particularly suitable for NPC due to the irregular target volume and proximity of critical structures (12). In the previous study, we observed satisfactory short-term efficacy, with 3-year loco-regional control rate and DMFS of more than 95%, and 3-year OS was more than 85% in 190 NPC patients treated with HT (13), this study with long-term follow-up showed that 10-year local and regional control remained at about 95% at 10 years, while DMFS reduced to 86%, and OS to 77.8%.

In a phase III trial including 408 patients with stage III/IV NPC, were treated with conventional 2-dimensional radiotherapy, the 10-year DFS, LRFS, and OS were 66.9%, 80.8% and 49.5%, respectively (8). Han et al. reported that the 10-year LRFS, DFS and disease-specific survival (DSS) for NPC patients with stage II-III, treated with IMRT technique, were 92%, 83.4% and 78.6% respectively (14). In the present study, the 10-year LRFS, NRFS, DMFS and OS were 94%, 95%, 86%, and 77.8%, respectively, which were significantly higher than those reported in the era of conventional RT. The reason of our better results may be related to the inclusion of stage I-II patients, with stage I accounting for 8%. In our study, the 10-year DMFS and OS of stage III-IV patients were 80% and 76%; respectively. The 10-year LRFS for patients with stage I-II was better than those with stage III-IV (98% vs. 92%, p= 0.04). In a phase III trial, 230 NPC patients with stage II achieved excellent results, with the 10-year DFS, PFS and OS of 94%, 76.7% and 83.6%; respectively (15). HT provides a technical platform to increase the dose in tumor target volume, and represents better local control (16). Belgioia et al. reported that 2 and 4-year loco-regional control rates were 92.9% and 88.2%, respectively, and 4-year OS was 93.9% in advanced NPC patients treated by HT with simultaneous integrated boost (SIB) technique, and with a dose of 66Gy/30F to the primary tumor (17).

In our research, although the 10-year NRFS and OS in patients with stage T3-4 were worse than those with stage T1-2 (*p<* 0.05), No statistical difference was detected in multivariate analysis (*p*>0.05). The 10-year LRFS and OS in patients with negative node were better than those with metastatic nodes, although without statistical significance (*p*>0.05). The 10-year NRFS and DMFS in patients with stage N2-3 were worse than those with stage N0-1 (*p<* 0.05), but no statistical difference was detected in multivariate analysis (*p*>0.05). A review showed, in 610 NPC patients with stage N0 undergoing definitive radiotherapy to their primary lesion and prophylactic radiation to upper neck, the 5-year and 10-year regional failure-free survival could be 95.8% and 91.8%; respectively (17). Han et al. demonstrated that omitting bilateral or contralateral lower neck radiotherapy would be safe and feasible for NPC patients with stage N0-1, with the potential to reduce late toxicities, is a direction of reducing toxicity and increasing efficiency (18).

Distant metastasis has become the most common mode of treatment failure. According to previous literature reports (14), the 10-year distant metastasis rate was 16.6% (144/865). In our study, there were 25 cases of distant metastases, and the rate of distant metastases was 13.2%, which was consistent with previous research results and even lower. An advanced N stage has been shown to be one of the risk factors to predicting the occurrence of metastasis (19). This raises the question of how to select patients at high risk of distant metastases who would benefit from individual therapy to improve their OS. The results of the this study indicated that the 10-year DMFS for patients with negative node were better than those with positive node. The factor of the clinical stage was also associated with the risk of distant metastasis. Patients with a more advanced stage, especially those with T4N+ disease with intracranial or neural invasion, have a higher risk of lymph node metastasis and distant metastasis. T and N stage factors were associated with distant metastasis, although without statistical significance (p>0.05) in multivariate analysis. This may mean that the difference between the treatment and the patient itself has become a confounding factor in the analysis.

In this study, 12, 8 and 25 patients had developed local failure, lymph nodal failure and distant metastasis; respectively. Lung, bone and live were the most common metastatic organs. In addition, Five patients (stage T3-4) died of primary pharyngeal vascular hemorrhage within one year after radiotherapy. Six patients still died of massive nasal and laryngopharyngeal hemorrhage in long-term follow-up after radiotherapy, and one patients died of cerebral hernia 9 months after the completion of radiotherapy, meaning that pharyngeal vessel bleeding (primary or secondary) was one of the leading cause of death. On the premise of ensuring the effect of tumor control, the radiation dose protection, nutrition, flushing and closer follow-up may reduce the occurrence of such events.

At present, radical radiation therapy alone is recommended to stage I NPC, additional chemotherapy with neoadjuvant, concomitant or adjuvant forms is recommended to loco-regionally advanced NPC (14). Meta-analyses showed that concomitant chemo-radiotherapy improves survival in patients with loco-regionally advanced NPC, but the specific benefits of adjuvant chemotherapy after concomitant chemo-radiotherapy needs further study (20). Blanchard et al. analyzed data from 19 trials and 4806 patients and found that chemotherapy improved absolute overall survival by 6.3% over 5 years, and also improved progression-free survival, loco-regional control, distant control and reduced cancer mortality significantly (*p* <0.0001). The benefit of concomitant with or without adjuvant chemotherapy to radiation therapy was significantly (*p*=0.01), but not adjuvant chemotherapy alone or neoadjuvant chemotherapy alone. In this study, the 10-year OS in patients with concomitant chemotherapy group was better than that in the non-chemotherapy group in multivariate analysis (p<0.05).

n recent years, anti-EGFR Mabs such as cetuximab and nimotuzumab were applied as a concomitant therapy with radiation therapy for head and neck cancer (21). Bonner et al. (22)reported significant improvement in survival in patients with non-NPC head and neck cancer when cetuximab was added to radiation therapy in a phase III trial. In the meta-analysis of Yuan et al. (23), anti-EGFR Mab combined with radiation therapy and/or chemotherapy improved the short-term therapeutic effect in NPC, but this benefit disappeared 1 year later. In this study, 81 patients (42.6%) had anti-EGFR Mab with HT and 30 (15.8%) with HT and chemotherapy, Unfortunately, only benefit in DMFS was detected, and without statistical significance (p>0.05). So the effect of anti-EGFR Mab combined with chemo-radiotherapy in NPC needs further investigations.

Huang et al. (24) reported that age and N stage were independent prognostic factors for OS, radiation dose was an independent prognostic factor for loco-regional control, and N stage was an independent prognostic factor for distant metastasis. In our update, age, concurrent chemotherapy and stage were independent prognostic factors for OS, which was consistent with the report.

Since the era of IMRT, the incidence of late grade 3 to 4 toxicities of xerostomia and neck fibrosis after radiotherapy was reduced to 16.7% and 8.3% (24). HT is a unique IMRT modality that can provide better conformity index, steeper dose gradient, shorter treatment time, and better protection of many organs at risk (OARs) especially for parotids (25).The acute treatment-related toxicities of HT in NPC treatment, which were relatively mild, In addition, the incidence of acute grade- 2 xerostomia was only 7.3% were described in our previous report (8). Belgioia et al. reported that the most significant acute toxicities were grade 2 or 3 mucositis (43%); grade 2 xerostomia was reported in 11 patients after 6 months from the end of treatment and downgrade to level 1 in 55% (6/11) patients within 12 months (6). At the last follow-up visit in our study, 139 out of 190 patients could be evaluated for the late treatment-related toxicities, xerostomia, subcutaneous tissue fibrosis, hearing loss, and tooth sensitivity were the most common late toxicities which were mainly scored as grade1 or 2. Two patients had tooth looseness, and 2 others pulpitis which needed surgical operation. It is worth noting that although the response to radiotherapy and chemotherapy is not significant, the incidence of massive bleeding complications of nasopharyngeal carcinoma in the follow-up study was 5.8%. Five cases occurred within one year after radiotherapy, and six cases occurred in long-term follow-up after radiotherapy. It suggests that the risk of major bleeding is still high for patients with late T stage and neck metastatic lymph node fusion surrounding blood vessels. During follow-up, attention should be paid to nasal cavity flushing and prevention.

There are several limitations of this study. First, the retrospective study affect the outcomes; second, patient distributions in our cohort were complex, I-IV, T1-4, N0-3 stages, which could affect the outcomes with confounding factors; third, synchronous and/or adjuvant chemotherapy, the nonuniformity pattern of combined chemotherapy could affect the outcomes of the study.

## Conclusions

Helical tomotherapy with simultaneous integrated boost technique offered excellent long-term outcomes for NPC patients, with mild late treatment-related toxicities. Age and clinical stage was independent predictor of DMFS and OS. Further prospective study is needed to confirm the superiority of this technology and to evaluate the roles of anti-EGFR monoclonal antibody treatment.

## Data availability statement

The original contributions presented in the study are included in the article/supplementary material. Further inquiries can be directed to the corresponding author.

## Ethics statement

This study was conducted with approval from the Ethics Committee of the Chinese PLA General Hospital. Written informed consent was obtained from all participants.

## Author contributions

LMe, FT, and QL made equal contributions to this work, participated in the design of the research, carried out research, made statistical analysis and drafted the manuscript. LMa conceived and designed the study and reviewed the manuscript. All authors read and approved the final manuscript.
